# Accuracy of preoperative clinical staging for locally advanced gastric cancer in KLASS-02 randomized clinical trial

**DOI:** 10.3389/fsurg.2022.1001245

**Published:** 2022-09-23

**Authors:** Dong Jin Kim, Woo Jin Hyung, Young-Kyu Park, Hyuk-Joon Lee, Ji Yeong An, Hyoung-Il Kim, Hyung-Ho Kim, Seung Wan Ryu, Hoon Hur, Min-Chan Kim, Seong-Ho Kong, Jin-Jo Kim, Do Joong Park, Keun Won Ryu, Young Woo Kim, Jong Won Kim, Joo-Ho Lee, Han-Kwang Yang, Sang-Uk Han, Wook Kim, on behalf of the Korean Laparoendoscopic Gastrointestinal Surgery Study (KLASS) Group

**Affiliations:** ^1^ Department of Surgery, Eunpyeong St. Mary's Hospital, College of Medicine, The Catholic University of Korea, Seoul, South Korea; ^2^ Department of Surgery, Yonsei University College of Medicine, Seoul, South Korea; ^3^ Department of Surgery, Chonnam National University Medical School, Gwangju, South Korea; ^4^ Department of Surgery and Cancer Research Institute, Seoul National University College of Medicine, Seoul, South Korea; ^5^ Department of Surgery, Samsung Medical Center, Sungkyunkwan University School of Medicine, Seoul, South Korea; ^6^ Department of Surgery, Seoul National University Bundang Hospital, Seongnam, South Korea; ^7^ Department of Surgery, Keimyung University Dongsan Medical Center, Daegu, South Korea; ^8^ Department of Surgery, Ajou University School of Medicine, Suwon, South Korea; ^9^ Department of Surgery, Dong-A University Hospital, Busan, South Korea; ^10^ Department of Surgery, Incheon St Mary's Hospital, The Catholic University of Korea, Incheon, South Korea; ^11^ Center for Gastric Cancer, National Cancer Center, Goyang, South Korea; ^12^ Department of Surgery, Chung-Ang University Hospital, Seoul, South Korea; ^13^ Department of Surgery, Nowon Eulji Medical Center, Eulji University, Seoul, South Korea; ^14^ Department of Surgery, Yeouido St Mary's Hospital, The Catholic University of Korea, Seoul, South Korea

**Keywords:** gastric neoplasm, diagnosis, accuracy, gastroscopy, computed tomography

## Abstract

**Purpose:**

The discrepancy between preoperative and final pathological staging has been a long-standing challenge for the application of clinical trials or appropriate treatment options. This study aimed to demonstrate the accuracy of preoperative staging of locally advanced gastric cancer using data from a large-scale randomized clinical trial.

**Materials and methods:**

Of the 1050 patients enrolled in the clinical trial, 26 were excluded due to withdrawal of consent (*n* = 20) or non-surgery (*n *= 6). The clinical and pathological staging was compared. Risk factor analysis for underestimation was performed using univariate and multivariate analyses.

**Results:**

Regarding T staging by computed tomography, accuracy rates were 74.48, 61.62, 58.56, and 85.16% for T1, T2, T3 and T4a, respectively. Multivariate analysis for underestimation of T staging revealed that younger age, ulcerative gross type, circular location, larger tumor size, and undifferentiated histology were independent risk factors. Regarding nodal status estimation, 54.9% of patients with clinical N0 disease were pathologic N0, and 36.4% of patients were revealed to have pathologic N0 among clinical node-positive patients. The percentage of metastasis involvement at the D1, D1+, and D2 lymph node stations significantly increased with the advanced clinical N stage. Among all patients, 29 (2.8%), including 26 with peritoneal seeding, exhibited distant metastases.

**Conclusions:**

Estimating the exact pathologic staging remains challenging. A thorough evaluation is mandatory before treatment selection or trial enrollment. Moreover, we need to set a sufficient case number when we design the clinical trial considering the stage migration.

## Introduction

Gastric cancer is the fifth most common cancer and the third most common cause of cancer-related deaths globally (1). In Korea, while the proportion of early gastric cancer has increased owing to the performance of biannual screening endoscopy, advanced gastric cancer still accounts for a considerable proportion of cases (2–4). Recently, various new procedures and treatment strategies have been developed and applied. These updates have been included in the guidelines (5–9). In addition, an increasing number of clinical trials are being performed for gastric cancer to improve treatment of the disease (8, 9).

The implementation of these treatment guidelines or decisions regarding patient enrollment in clinical trials depends on the preoperative or pretreatment clinical diagnosis. The decision regarding the treatment algorithm to pursue, enroll in, or drop out from clinical studies is based on gastrofiberoscopy (GFS) or computed tomography (CT) images (5, 10, 11). Primarily, preoperative estimation of tumor depth is conducted using GFS, endoscopic ultrasonography (EUS), and CT images. The consensus for the role of each modality is that GFS differentiates between early and advanced gastric cancer, whereas EUS or CT are used to estimate the depth of invasion more specifically. However, the accuracy of the tumor depth estimation for each diagnostic modality has not yet been established.

Matters concerning correct diagnosis, as well as more critical issues directly related to patient care, such as overtreatment that may cause additional harm to the patient and undertreatment, which might result in incomplete treatment and early treatment failure, are critical.

In the historic MAGIC clinical trial regarding perioperative chemotherapy, patients were assigned to the surgery-only or perioperative chemotherapy groups (12). Among 253 patients in the surgery group, 8.3% were diagnosed with pathologic T1 cancer. This result indicates that approximately 8.3% of the patients in the perioperative chemotherapy group might have undergone unnecessary preoperative chemotherapy. Similar results were reported in recent clinical trials. The KLASS-01 trial compared the 5-year overall survival between laparoscopic distal gastrectomy and open distal gastrectomy for clinical stage I gastric cancer. In the final pathologic diagnosis, 197 of the 1,359 intention-to-treat groups (14.5%) had stage II or more advanced disease (11). KLASS-02 trial was the randomized controlled trial which designed to reveal the non-inferiority of laparoscopic distal gastrectomy over open distal gastrectomy regarding the 3 year relapse free survival for locally advanced gastric cancer (13, 14). The study was well designed, and reported morbidity and mortality data ahead of further follow up for the result of primary end point (15). In spite of the inclusion criteria of KLASS-02, 25.7% of patients were diagnosed with early gastric cancer (10). Similar problems have also been shown in the Chinese trial (CLASS-01). In that trial, 29.2% of patients were diagnosed with early gastric cancer, even though preoperative advanced gastric cancer patients were enrolled (16).

Discrepancies remain between preoperative clinical staging and final diagnosis. This study aimed to explore the current diagnostic accuracy using prospectively collected data from the KLASS-02 randomized clinical trial. In addition, we attempted to define clinical T and N staging, as well as the risk factors for preoperative underestimation.

## Materials and methods

### Design of original study and participants

The KLASS-02-RCT was an investigator-initiated phase III, multicenter, open-label, prospective randomized trial conducted by 20 surgeons from 13 university hospitals in South Korea (10). KLASS-02 trial was conducted to confirm the non-inferiority of laparoscopic gastrectomy compared to open gastrectomy in locally advanced gastric cancer.

The indications for the study were confined to clinical stages T2, T3, and T4a with respect to tumor depth. In cases of discrepancies in the results of tumor depth estimation among the diagnostic modalities, more advanced findings were the rationale for deciding to enroll patients. Therefore, clinical T1 patients can be included in comparative results using each diagnostic modality. Regarding lymph node status, clinical N0 or clinically node-positive confined to the perigastric area was indicated for this study. Patient enrollment was comprehensively decided based on the results of GFS, EUS, and CT.

**Figure 1:**
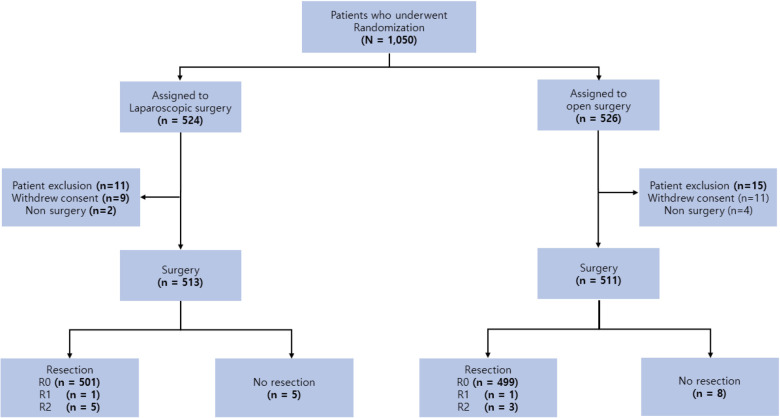
Schema showing npatient selection.

Among the 1,050 patients enrolled, 524 were assigned to laparoscopic distal gastrectomy and 526 to open distal gastrectomy. In the current study, all patients who were not eligible to confirm the tumor depth, lymph node status, or metastatic status were excluded; finally, 1,024 patients were included, which was not the same group as the original paper for the primary endpoint (Figure 1). This retrospective study was approved by the Institutional Review Board of our institution (IRB number: SC21RIDI0054).

### Standardization of the radiologic evaluation for tumor staging

The mandatory evaluation tools included the GFS and CT. EUS is optional for tumor depth evaluation. The GFS and EUS findings were completely dependent on the gastroenterologist. In the case of CT, we abided by the radiologists' decisions regarding the tumor depth and lymph node status. Preoperative stomach computed tomography (CT) was performed with gastric distension using gas vaporization or contrast water. No additional consensus meetings were held among radiologists working at the participating institutions involved in this trial. Rather, all radiologic evaluations were performed based on consensus among the radiologic societies (17). (T1a—tumor showed enhancement and/or thickening of the abnormal mucosa, compared to the adjacent normal mucosa, with an intact low-density stripe; T1b, disruption of the low-density stripe (<50% of the thickness); T2, disruption of the low-density stripe (>50% of the thickness) without abutting the outer high-attenuating layer; T3, discrimination between the enhancing gastric lesion and the outer layer was indiscernible, and a smooth outer margin of the outer layer or a few small linear stranding in the perigastric fat plane were visualized; T4a, an irregular or nodular outer margin of the outer layer and/or a dense bandlike perigastric fat infiltration was visualized; and T4b, obliteration of the fat plane between the gastric lesion and the adjacent organs or direct invasion of the adjacent organs).

Regarding nodal status, while there are no definite criteria for setting the clinical N-stage by CT imaging, deciding on the clinical N-stage was also at the discretion of the radiologist and was regarded as a metastatic node if the short diameter of the lymph node size exceeded 8 mm (18).

### Data management and analysis plan

All data analyses were conducted according to the guidelines of the American Joint Committee on Cancer (AJCC) 8th edition. During this clinical study, data were collected according to the AJCC on Cancer 7th edition guidelines. All data were revised to a new edition and analyzed.

One patient classified as having near subtotal distal gastrectomy was recategorized as having distal gastrectomy for the accuracy of data classification.

Regarding tumor location in the circular direction, data were revised into four types: lesser curvature, greater curvature, anterior or posterior walls, or circular location, defined as tumor confined to more than three areas among the anterior, posterior, lesser curvature, or greater curvature. Among the various histological types, papillary carcinoma and well-to-moderately differentiated adenocarcinoma were classified as differentiated types, and poorly differentiated adenocarcinoma, signet ring cell carcinoma, mucinous carcinoma, and undifferentiated type were classified as undifferentiated types. The gross shape of the tumor was divided into depressed and non-depressed groups. The depressed type included early gastric cancer types IIc, III, Borrmann type II, III, and IV, and the others were classified as the undepressed type.

Clinical and pathologic T stages were compared using a simple cross-table description. The clinical *T*-stage was evaluated using CT and EUS. Regarding *T*-stage estimation, various clinicopathological characteristics were used to define the risk factors for underestimation, which means that the final pathologic result was shown to be more advanced than clinical estimation. Cases in which the final pathological depth could not be defined were excluded. Multivariate analysis was performed for the factors that were significant in the univariate analysis. Clinical N stage and pathological N state were compared according to N0, N1, N2, and N3, respectively. Additionally, the distribution of metastatic lymph nodes in the D1, D1+, and D2 areas was compared according to clinical nodal status. The sensitivity, specificity, positive and negative predictive values, and accuracy rates were calculated for each T and N stage within the cases that were precisely defined for the pathologic T and N stages. Each diagnostic ability parameter was defined as follows in case of T1: sensitivity, cT1/pT1; specificity, (cT2, T3, T4a)/(pT2, T3, T4a); positive predictive value (PPV) (pT1/cT1); negative predictive value (NPV) (pT2, T3, T4a)/(cT2, T3, T4a); and accuracy rate (cT1 / pT1 + cT2,3,4a / pT2,3,4a)/total number of patients in each analysis. Patients with unknown pathological depth or lymph node metastasis status were excluded from analysis.

The presence of distant metastatic lesions according to each clinical TNM staging and location of metastasis was analyzed and described.

### Statistical analyses

A general descriptive analysis was performed. Continuous variables are expressed as mean ± standard deviation, whereas nominal variables are expressed as numbers and percentages. In the risk factor analysis for underestimation, the Student's *t*-test and chi-square analysis were performed for continuous and nominal variables, respectively. A multivariate analysis was performed using a logistic regression model. Analysis of the distribution of metastatic lymph nodes and peritoneal metastasis according to clinical N and T stages was performed using the chi-square test. Statistical significance was set at *p* < 0.05. All statistical analyses were performed using the PASW software (SPSS Inc., Chicago, IL, USA).

## Results

Regarding T staging compared between pathologic T stage and clinical T stage estimated by CT, 41.5, 27.7, and 30.3% of clinical T2 cases were classified as under T2, T2, and over T2 cases, respectively (Table 1). Among the clinical T3 cases, 17.6, 21.9, 31.1, and 28.6% of patients had T1, T2, T3, and T3, respectively. Among the clinical T4a cases, 7.6% of the patients had early gastric cancer, and 44.5% of the patients exhibited the same stage as the clinical estimation. The EUS results were available for 422 patients. Among the 215 patients estimated with depth of invasion of the proper muscle, 52 patients (24%) were diagnosed with pathologic T2, 85 patients (40%) had early gastric cancer, and the other 77 patients (36%) were diagnosed with T2. Among the 125 clinical T3 patients diagnosed by EUS, pathologic T3 cases were the most common, exhibited by 42 patients (34%). Twelve patients (9%) had early gastric cancer, and 29 (23%) had pathologic stage T2.

**Table 1:** Discrepancy between clinical T by computed tomography and pathologic depth.

Variables	pT1	pT2	pT3	pT4a	pT4b	pTx	Total
**cT1**	22 (62.9%)	7 (20.0%)	4 (11.4%)	2 (5.7%)	0 (0.0%)	0 (0.0%)	35 (100%)
**cT2**	160 (41.5%)	107 (27.7%)	78 (20.2%)	39 (10.1%)	0 (0.0%)	2 (0.5%)	386 (100%)
**cT3**	69 (17.6%)	86 (21.9%)	122 (31.1%)	109 (27.8%)	3 (0.8%)	3 (0.8%)	392 (100%)
**cT4a**	16 (7.6%)	18 (8.5%)	70 (33.2%)	94 (44.5%)	5 (2.4%)	8 (3.8%)	211 (100%)
**Total**	**267** **(26.1%)**	**218** **(21.3%)**	**274** **(26.8%)**	**244** **(23.8%)**	**8** **(0.8%)**	**13** **(1.3%)**	**1,024** **(100%)**

Values are expressed with number and percentage.

In the univariate analysis to identify risk factors for underestimation during the preoperative evaluation, younger age, ulcerative gross features, tumor location with circular features, larger tumor size, undifferentiated histology type, and higher CA19–9 levels were identified as risk factors. In multivariate analysis, age (0.987 [0.974–1.000]; *p* = 0.044), ulcerative gross shape (OR: 2.574 [1.531–4.329]; *p* < 0.001), circular location (2.250 [1.140–4.439], 0.019), tumor size (OR: 1.182 [1.105–1.264], *p* < 0.001), and undifferentiated histology (OR: 1.704 [1.273–2.280, *p* < 0.001]) were risk factors (Table 2).

**Table 2:** Univariate and multivariate risk factor evaluation for underestimation for T stage.

Variables	Equal or overestimation	Underestimation	*p*	OR (95% CI)	*p*
Sex	Male	306 (42.3%)	417 (57.7%)	0.399		
	Female	118 (39.5%)	181 (60.5%)			
Age (year)		60.7 ± 10.8	58.8 ± 11.7	**0** **.** **007**	0.987 (0.974–1.000)	**0** **.** **044**
BMI (kg/m2)		23.5 ± 3.2	23.6 ± 3.1	0.607		
ASA	1	190 (38.5%)	304 (61.5%)	0.165		
	2	212 (44.4%)	266 (55.6%)			
	3	22 (44.0%)	28 (56.0%)			
Gross type	EGC type I	4 (80.0%)	1 (20.0%)	**<0** **.** **001**		
	EGC type IIa	14 (60.9%)	9 (39.1%)			
	EGC type IIb	2 (100.0%)	0 (0%)			
	EGC type IIc	19 (61.3%)	12 (38.7%)			
	EGC type III	6 (42.9%)	8 (57.1%)			
	AGC Borrmann-I	37 (64.9%)	20 (35.1%)			
	AGC Borrmann-II	71 (35.7%)	128 (64.3%)			
	AGC Borrmann-III	267 (39.9%)	402 (60.1%)			
	AGC Borrmann-IV	4 (19.0%)	17 (81.0%)			
	AGC Borrmann-V	0 (0%)	1 (100%)			
Gross type 2	Non-depressed	57 (65.5%)	30 (34.5%)	**<0** **.** **001**	Reference	
	Ulcerative type	367 (39.3%)	568 (60.7%)		2.574 (1.531–4.329)	**<0** **.** **001**
Tumor location	Lesser curvature	145 (44.2%)	183 (55.8%)	**0** **.** **004**	Reference	
	Greater curvature	78 (39.4%)	120 (60.6%)		1.185 (0.805–1.745)	0.390
	Anterior or posterior	158 (43.5%)	205 (56.5%)		1.081 (0.782–1.495)	0.637
	Circular	13 (20.6%)	50 (79.4%)		2.250 (1.140–4.439)	**0** **.** **019**
Tumor size (cm)		4.12 ± 2.18	5.02 ± 2.57	**<0** **.** **001**	1.182 (1.105–1.264)	**<0** **.** **001**
Differentiation	Differentiated	211 (52.8%)	189 (47.3%)	**<0** **.** **001**	Reference	
	Undifferentiated	204 (34.9%)	381 (65.1%)		1.704 (1.273–2.280)	**<0** **.** **001**
CEA (ng/ml)		3.98 ± 16.91	3.77 ± 16.07	0.843		
CA19-9 (U/ml)		13.95 ± 24.78	31.59 ± 132.68	**0** **.** **002**	1.004 (1.000–1.008)	0.062

Continuous variables are described as mean ± standard deviation, and nominal variables are described as number and percentage.

BMI, body mass index; ASA, American Society of Anesthesiology; EGC, early gastric cancer; AGC, advanced gastric cancer; CEA, carcinoembryonic antigen.

**Figure 2:**
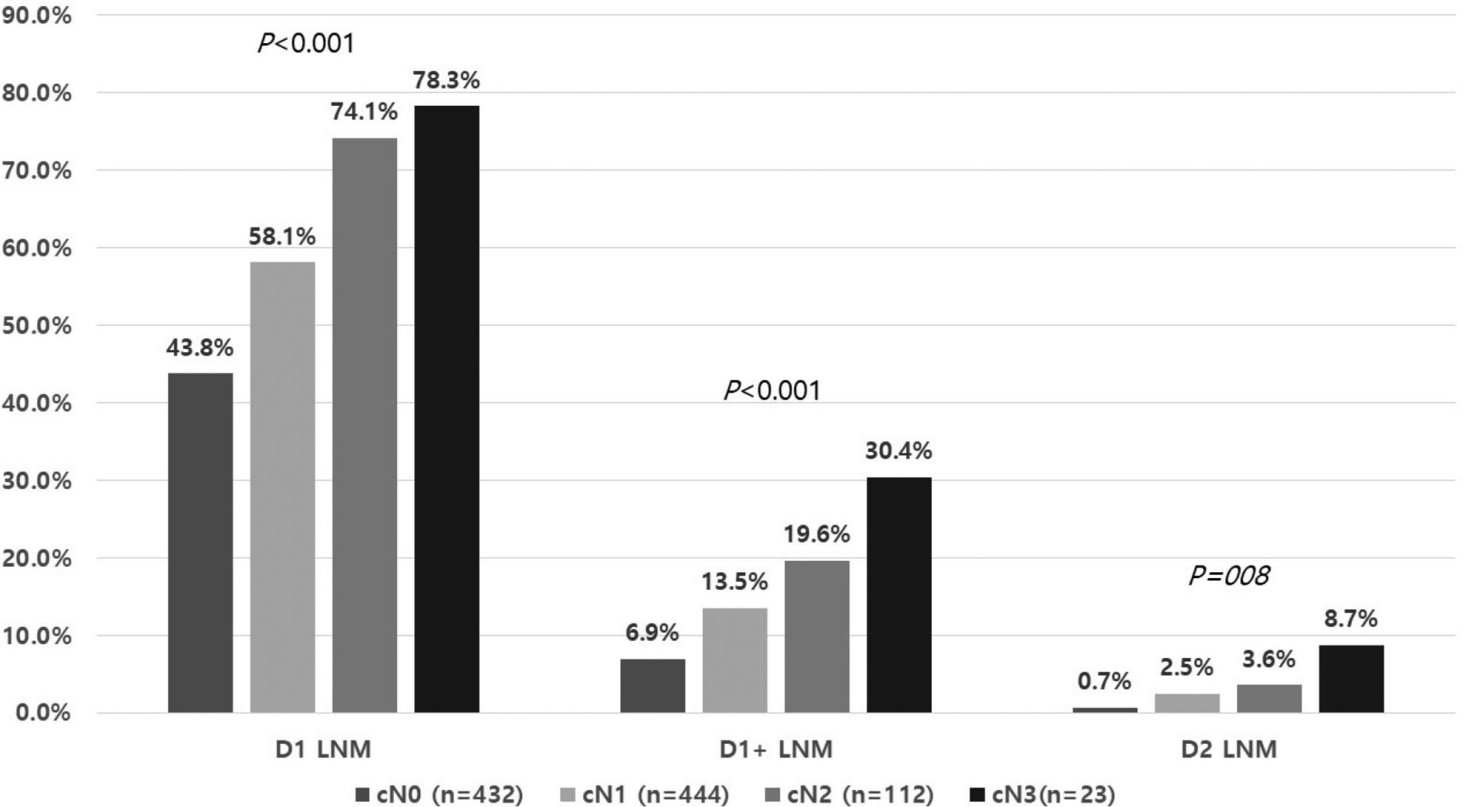
Rate of lymph node metastasis in each lymph node station according to clinical N stage.

Regarding nodal status estimation, 54.9% of patients with clinical N0 disease were pathologic N0, and 36.4% of patients were identified as having pathologic N0 among the clinical node-positive patients (Table 3). In the distribution analysis of metastatic lymph nodes according to clinical nodal status, the percentage of lymph node metastasis significantly increased with the severity of the clinical nodal stage. For lymph node metastasis in the D1 area, 43.8, 58.1, 74.1, and 78.3% of patients had D1 area metastatic lymph nodes in the cN0, cN1, cN2, and cN3 groups, respectively. For the D2 LNM area, 0.7, 2.5, 3.6, and 8.7% of patients had metastatic lymph nodes in the D2 lymph node area in cN0, cN1, cN2, and cN3 patients, respectively (Figure 2).

**Table 3:** Discrepancy between clinical N stage and pathologic N stage.

	pN0	pN1	pN2	pN3a	pN3b	Total
**cN0**	234 (54.9%)	84 (19.4%)	63 (14.6%)	37 (8.6%)	11 (2.5%)	432 (100%)
**cN1**	178 (40.1%)	86 (19.4%)	80 (18.0%)	64 (14.4%)	36 (8.1%)	444 (100%)
**cN2**	28 (25.0%)	27 (24.1%)	17 (15.2%)	28 (25.0%)	12 (10.7%)	112 (100%)
**cN3**	5 (21.7%)	2 (8.7%)	3 (13.0%)	9 (39.1%)	4 (17.4%)	23 (100%)
**Total**	448 (44.3%)	199 (19.7%)	163 (16.1%)	138 (13.6%)	63 (6.2%)	1,011 (100%)

Values are expressed with number and percentage.

The sensitivity, specificity, positive and negative predictive values, and accuracy rates were calculated for each T and N stage (Table 4).

**Table 4:** Sensitivity, specificity, positive and negative predictive values, and accuracy rate for each T and N stage.

Variable	Computed tomography				Endoscopic ultrasonography				Computed tomography			
	T1	T2	T3	T4a	T1	T2	T3	T4a	N0	N1	N2	N3
Sensitivity	8.24	49.08	44.53	38.52	13.71	54.74	36.52	20.93	52.23	43.22	10.43	15.12
Specificity	98.25	65.07	63.09	85.79	95.55	49.53	73.42	90.00	64.83	55.91	88.80	98.92
PPV	62.86	27.72	31.12	44.55	56.67	24.30	34.43	35.29	54.17	19.37	15.18	56.52
NPV	73.91	80.88	74.37	80.57	72.28	78.71	75.17	81.37	63.04	80.07	83.76	92.61
Accuracy rate	74.48	61.62	58.56	85.16	71.15	50.72	63.22	75.72	59.25	53.41	76.16	91.79

All values are expressed with percentage (%).

PPV, positive predictive value, NPV, negative predictive value.

Among all the patients, 29 (2.8%) had distant metastases. Peritoneal metastasis was the most common metastatic site and was confined to 26 patients. Retroperitoneal metastatic lymph nodes and hepatic metastasis were observed in two and one patient, respectively. The detailed clinical stages of patients with peritoneal metastasis are presented in Table 5. There was a significant increase in the incidence of peritoneal metastasis according to clinical *T*-stage (*p* = 0.02).

**Table 5:** Frequency of peritoneal metastasis according to the clinical stage.

cStage	Peritoneal metastasis	Total number of patients	*p*
**cT1**	cT1N0	0	30	0.02
** **	cT1N1	0	5	
** **	Total	0	35	
**cT2**	cT2N0	3 (1.3%)	230	
** **	cT2N1	2 (1.5%)	136	
** **	cT2N2	0 (0%)	17	
** **	cT2N3	0 (0%)	3	
** **	Total	5 (1.3%)	386	
**cT3**	cT3N0	1 (0.7%)	134	
** **	cT3N1	6 (2.5%)	203	
** **	cT3N2	1 (1.9%)	52	
** **	cT3N3	0 (0%)	3	
** **	Total	8 (2%)	392	
**cT4**	cT4aN0	1 (2.6%)	39	
	cT4aN1	8 (7.3%)	109	
	cT4aN2	4 (8.7%)	46	
	cT4aN3	0 (0%)	17	
	Total	13 (6.2%)	211	

**Figure 3:**
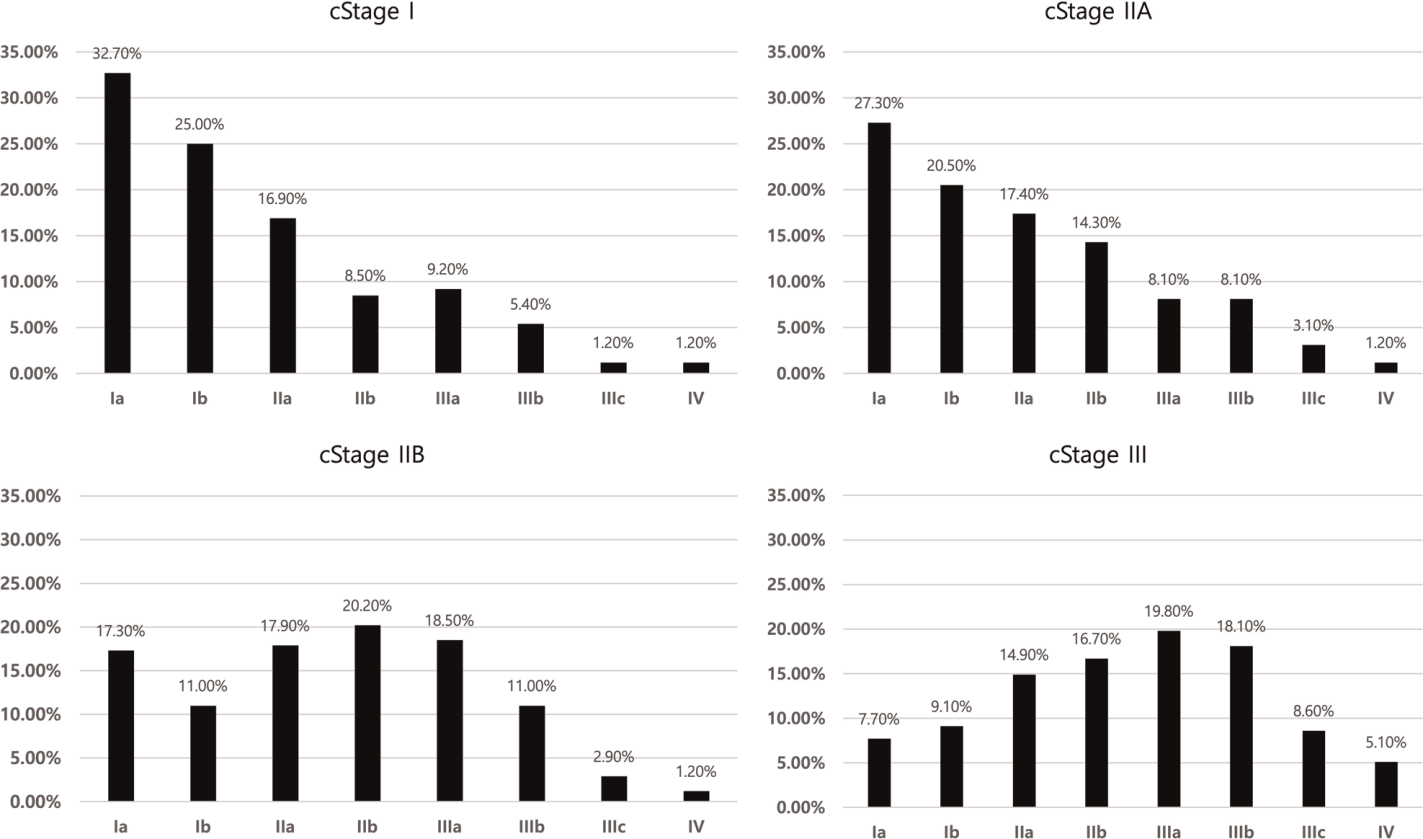
Distribution of pathologic staging among clinical stages according to 8th AJCC TNM staging system.

The final pathological stage distribution according to clinical staging based on the AJCC 8th edition is shown in Figure 3. Clinical stage IIA includes more pathological stage I patients and has poor discrimination ability compared to clinical stage I. Clinical stage IIB includes the highest proportion of pathological stage IIB. Among clinical stage III cases, pathological stage IIIA was the most common, whereas 16.8% were stage I and 5.1% were stage IV. With the result of the 8th AJCC staging system, risk evaluation for underestimation was done similarly with *T*-staging (Table 6). Excluding stage IV patients, circular gross type tumor and undifferentiated tumor were revealed as risk factors for underestimation regarding the 8th AJCC staging system. Sub-analysis with 217 patients who were underestimated as TNM staging, 137 (63.1%) and 131 (60.4%) patients were involved with underestimation for *T*-stage and N-stage, respectively.

**Table 6:** Univariate and multivariate risk factor evaluation for underestimation for clinical stage according to 8th AJCC classification.

Variables	Equal or overestimation	Underestimation	*p*	OR (95% CI)	*p*
Sex	Male	560 (72.0%)	143 (65.9%)	0.082		
	Female	218 (28.0%)	74 (34 0.1%)			
Age (year)		60.0 ± 11.0	58.5 ± 12.4	**0** **.** **098**	0.991 (0.976–1.007)	0.266
BMI (kg/m2)		23.5 ± 3.2	23.8 ± 2.9	0.607		
ASA	1	337 (48.5%)	103 (47.5%)	**0** **.** **050**	Reference	
	2	357 (45.9%)	110 (50.7%)		1.289 (0.915–1.816)	0.146
	3	44 (5.7%)	4 (1.8%)		0.237 (0.055–1.014)	0.052
Gross type	EGC type I	5 (0.6%)	0 (0%)	0.329		
	EGC type IIa	19 (2.4%)	4 (1.8%)			
	EGC type IIb	2 (0.3%)	0 (0%)			
	EGC type IIc	25 (3.2%)	6 (2.8%)			
	EGC type III	10 (1.3%)	4 (1.8%)			
	AGC Borrmann-I	49 (6.3%)	8 (3.7%)			
	AGC Borrmann-II	145 (18.6%)	50 (23.0%)			
	AGC Borrmann-III	508 (65.3%)	141 (65.0%)			
	AGC Borrmann-IV	15 (1.9%)	3 (1.4%)			
	AGC Borrmann-V	0 (0%)	1 (0.5%)			
Gross type2	Non-depressed	75 (9.6%)	12 (5.5%)	**0** **.** **058**	Reference	
	Ulcerative type	703 (90.4%)	205 (94.5%)		2.574 (1.531–4.329)	0.166
Tumor location	Lesser curvature	264 (36.2%)	56 (28.3%)	**0** **.** **050**	Reference	
	Greater curvature	149 (20.4%)	41 (20.7%)		1.279 (0.809–2.023)	0.292
	Anterior or posterior	277 (38.0%)	82 (41.4%)		1.361 (0.926–2.001)	0.116
	Circular	39 (5.3%)	19 (9.6%)		2.150 (1.146–4.032)	**0** **.** **017**
Tumor size (cm)		4.55 ± 2.38	4.75 ± 2.51	0.275		
Differentiation	Differentiated	332 (42.7%)	64 (29.5%)	**<0** **.** **001**	Reference	
	Undifferentiated	446 (57.3%)	153 (70.5%)		1.652 (1.161–2.351)	**0** **.** **005**
CEA (ng/ml)		4.12 ± 18.60	3.14 ± 5.24	0.445		
CA19-9 (U/ml)		24.75 ± 112.4	21.88 ± 61.03	0.720		

Continuous variables are described as mean ± standard deviation, and nominal variables are described as number and percentage.

BMI, body mass index; ASA, American Society of Anesthesiology; EGC, early gastric cancer; AGC, advanced gastric cancer; CEA, carcinoembryonic antigen.

## Discussion

The diagnostic accuracy of KLASS-02 RCT was comprehensively examined in this study. As the indication for enrollment was confined to locally advanced gastric cancer, full exploration of all clinical stages was limited. However, the current diagnostic accuracy for clinically advanced gastric cancer in South Korea needs to be addressed. Furthermore, the results of this study can aid in guiding surgical strategies and clinical studies. This study is valuable in that it was a large-scale prospective randomized clinical trial in which patient enrollment was thoroughly considered in clinical diagnosis. Therefore, the clinical stage was collected relatively accurately compared with that of retrospective studies.

Regarding *T*-stage estimation by CT, most of the sensitivities and PPV, except T1 for PPV, were< 50% between the clinical stage and pathologic staging. With EUS, except for the sensitivity for T2 and PPV for T1, the sensitivity and PPV for each clinical tumor depth were< 50%. The accuracy rates for each clinical tumor depth determined using CT were 74.48, 61.62, 58.56, and 85.16% for T1, T2, T3, and T4a, respectively. The accuracy rates of EUS were 71.15, 50.72, 63.22, and 75.72% for T1, T2, T3, and T4a, respectively. In this analysis, clinical T1 cases were included because enrollment was decided on the basis of comprehensive CT and endoscopy or EUS findings. Therefore, some patients with clinical T1 cancers according to CT or EUS were enrolled and included in the analysis. Estimating the exact tumor depth is the most important factor when attempting to discriminate subjects for endoscopic submucosal dissection (19, 20). As such, in a situation like the KLASS-02 trial, overestimation can be problematic because excessive enrollment of early gastric cancer patients might weaken the power of the clinical trial. In addition, overtreatment, such as total omentectomy and mandatory D2 lymph node dissection for pathologically early gastric cancer, might increase the operation time and risk after gastrectomy (21, 22). Moreover, in clinical trials that use preoperative chemotherapeutic agents, inaccurate preoperative staging may cause patients to undergo unnecessary chemotherapy and additional harm (12).

A previous study evaluated the diagnostic performance of 64-section CT by two radiologists using CT gastrography-reviewed CT images of 127 patients (17). That study reported that the accuracies of *T*-stage estimation were 77.2 and 82.7%, respectively. This performance was similar to or superior to that of our results. However, the study included all clinical stages, including early gastric cancer, and only a small number of patients had extraordinary cases. The diagnostic accuracy for T1 cancer was higher than that for advanced gastric cancer. Therefore, it would be inappropriate to compare other retrospective studies including all clinical stages with the current study, which was confined to cases of locally advanced gastric cancer.

Overestimations and underestimations are important. In fact, the risk factors for overestimation were evaluated in this study, but no significant risk factors were identified. Underestimations may be related to undertreatment and early recurrence. In this study, we identified the independent risk factors for underestimation. These risk factors include younger age, ulcerative gross shape, larger tumor size, and undifferentiated histology. Several studies have sought to identify risk factors for underestimation. In another study, upper tumor location, tumor size> 2 cm, total gastrectomy compared with distal gastrectomy, and adjuvant chemotherapy were independent risk factors for pathologic advanced gastric cancer among clinical early gastric cancers (23). It is difficult to directly compare studies because our current study was designed to enroll patients with locally advanced gastric cancer. As the KLASS-02 trial was indicated for patients who were susceptible to distal gastrectomy, tumor location regarding proximal and distal issues was not included.

In the period during which this trial was designed, the role of EUS in estimating the depth of invasion was not promising and EUS evaluation was not mandatory. Only 431 of the 1,024 patients performed EUS. No improvement in the diagnostic quality was observed in the EUS results. Lee et al. studied the role of EUS to improve the accuracy of clinical T staging by CT (24). In their study, *T*-stage was classified into T1–2 and T3–4. The PPV and overall accuracy rates for T3–4 with CT only were 73.8 and 73.2%, respectively. Additional EUS information for T3–4 increased the PPV and overall accuracy to 85.3, and 74.8%, respectively. Recently, additional efforts have been made to overcome the limitations of the *T*-staging estimation. Magnetic resonance imaging with machine learning algorithms has also been used to improve diagnostic ability, but this study is still experimental (25).

Estimating the exact nodal stage is difficult when comparing clinical and pathological staging. We observed that 54.9% of the clinical N0 patients had pathologic N0 cases. Among the clinical N1 patients, 40.1% had pathological N0 disease. The clinical staging criteria for N-stage gastric cancer have not yet been standardized. Generally, lymph nodes with a short diameter (>8 mm) are regarded as positive lymph nodes (18). However, actual lymph size evaluation revealed that a large proportion of metastatic lymph nodes were< 8 mm (26). In a recent single-center study, the accuracy of the preoperative diagnosis of lymph node metastasis in gastric cancer showed an overall sensitivity of 44.4% and specificity of 93.4% (27). In general, because small metastatic lymph nodes are not detectable on CT scans, sensitivity is relatively lower than specificity.

In fact, clinical nodal status may not be important among clinical advanced gastric cancers under the current guidelines (5, 7). According to the current guidelines, the extent of lymph node dissection should be similar to that of D2 lymph node dissection. However, there is room for discussion regarding minimizing the extent of lymph node dissection (28, 29). These issues are related to efforts to achieve an optimal balance between oncologic curability, minimize postoperative complications, and impact on patients' quality of life. In the current study, we revealed that the exact estimation of the pathologic N stage was not promising using the current diagnostic approach. A recent retrospective study also showed the difficulty in estimating the exact N stage using only CT (30). However, we can find value in using the clinical N stage because there was a definite tendency for a wide range of lymph node metastases with advanced clinical nodal status in the current study.

Although all patients were enrolled after confirming the absence of distant metastasis, we identified 29 (2.8%) patients with distant metastasis. This value might be relatively higher than that reported in other studies because our study was indicated for clinically locally advanced gastric cancer. Among the 29 patients with stage IV cancer, 26 had peritoneal metastasis. Peritoneal seeding has been observed at various clinical stages. In summary, the rates of peritoneal seeding according to the clinical T stage were T1 0/35(0%), T2 5/386 (1.3%), T3 8/392 (2%) and T4a 13/211 (6.2%). There was a significant change in the rate of peritoneal metastasis when clinical tumor depth increased (*p* = 0.002). The current results highlight the role of diagnostic laparoscopy in determining the treatment strategy for advanced gastric cancer. One meta-analysis reported that 8.5%–59.6% of patients experienced alteration of their treatment after laparoscopic diagnosis (31). Nowadays, the laparoscopic approach for advanced gastric cancer is widely used. A thorough evaluation of the intra-abdominal space should be performed before performing the main gastrectomy procedure. In neoadjuvant cases, diagnostic laparoscopy should be mandatory to make the pre-treatment diagnosis more accurate (32).

The general consensus staging system used in this study was the AJCC 7th edition. In the AJCC 7th edition, there is no concept of clinical comprehensive staging, which was first introduced in the AJCC 8th edition and divided by cStages I, IIa, IIb, and III. We modified the current study data to the AJCC 8th edition system and explored the pathological staging distribution according to clinical staging. Pathological stage IA was the most common in both clinical stages I and IIA. However, pathological stages IIB and IIIA were the most common in clinical stage IIB and III patients, respectively. We observed a definite tendency for stage distribution according to clinical staging. Risk factor analysis for underestimation regarding AJCC 8th edition revealed the circular shape and undifferentiated type tumor were independent risk factors. The number of risk factors was decreased compared to the risk factors for underestimation of the T stage. This change might have arisen from a combination of the N-staging system. As the number and rate of underestimation patients regarding T and N-stage were similar, the power of both factors on the underestimation might be same.

The current study had some limitations. First, although the data were collected from multicenter tertiary hospitals with considerable gastric cancer treatment experience, considerable time has elapsed between data collection and the current period. This gap could influence the accuracy of estimating pathologic staging due to differences in the resolution of diagnostic images and improvements in interpretation.

The second problem is the bias from the diagnostic interpretation by radiologists or pathologists. Because this trial was conducted in 13 university hospitals, there might be the interpretation gap among hospitals. Unfortunately, we cannot estimate those discrepancies. For further accurate study, consensus meeting or cross-check activities are needed for minimizing those problems.

The third issue involved patient enrolment. Both the GFS and CT were sufficient for study enrollment to fulfill the inclusion criteria regarding clinical depth. This could have contributed to the relatively high number of patients with pathologic early gastric cancer in the final result. This may explain why some clinical T1 images were included in this study. In addition, the final decision for patient enrollment was at the discretion of the surgeon if the description of clinical depths was described as “cT1 or cT2″.

The accuracy rates for T2, T3, and T4a were 61.62%, 58.56%, and 85.16%, respectively. However, estimating the exact pathological stage remains challenging. Thorough evaluation is mandatory before treatment selection or trial enrolment. Moreover, we need to set a sufficient case number when designing a clinical trial considering stage migration.

## Contribution to the field statement

Diagnostic accuracy is crucial component for deciding appropriate surgical plan or patient enroll in clinical trials. Studies dealt with the subject are mostly retrospective studies. Current study used high quality data from KLASS-02 randomized clinical study which compared 3-year relapse free survival between laparoscopic and open distal gastrectomy for locally advanced gastric cancer. The result of this study would be helpful for the most surgeons and clinicians when they make decision for gastric cancer patients in their practice.

## Data Availability

The datasets presented in this article are not readily available because Dataset is controlled by the main PI of the KLASS-02 trial. The current study is for one of the collateral studies. Requests to access the datasets should be directed to Dong Jin Kim, djdjcap@catholic.ac.kr

## Authors disclaimer

Woo Jin Hyung – Employment: Hutom, Leadership: Hutom, Stock and Other Ownership Interests: Hutom Consulting or Advisory Role: Ethicon, Medtronic, and SK Hynix, Surgical Research Funding: Medtronic, GC Pharma. Han-Kwang Yang – Research Funding: Stryker; Hoon Hur, Honoraria: Johnson / Johnson Medical Research Funding: AstraZeneca, Dong-A ST. Do Joong Park – Research Funding: Medtronic, Daewoong Pharmaceutical. Jong Won Kim – Research Funding: Novomics, Daewoong Pharmaceutical. Sang-Uk Han – Research Funding: Jeil Pharmaceutical, Ethicon Endo-Surgery, Johnson / Johnson.
